# Effect of Graphene Flakes Modified by Dispersion in Surfactant Solutions on the Fluorescence Behaviour of Pyridoxine

**DOI:** 10.3390/ma11060888

**Published:** 2018-05-25

**Authors:** Rocío Mateos, Alba García-Zafra, Soledad Vera-López, María Paz San Andrés, Ana María Díez-Pascual

**Affiliations:** 1 Department of Analytical Chemistry, Physical Chemistry and Chemical Engineering, Faculty of Sciences, Alcalá University, 28871 Alcalá de Henares, Madrid, Spain; 2 Institute of Chemistry Research, “Andrés M. del Río” (IQAR), University of Alcalá, Ctra. Madrid Barcelona, Km. 33.6, 28871 Alcalá de Henares, Madrid, Spain; rocio.mateosm@edu.uah.es (R.M.); albagzaf@gmail.com (A.G.-Z.); soledad.vera@uah.es (S.V.-L.); mpaz.sanandres@uah.es (M.P.S.A.)

**Keywords:** pyridoxine, fluorescence, graphene, interaction, surfactant

## Abstract

The influence of graphene (G) dispersions in different types of surfactants (anionic, non-ionic, and cationic) on the fluorescence of vitamin B_6_ (pyridoxine) was studied. Scanning electron microscopy (SEM) was used to evaluate the quality of the G dispersions via measuring their flake thickness. The effect of surfactant type and concentration on the fluorescence intensity was analyzed, and fluorescence quenching effects were found for all of the systems. These turn out to be more intense with increasing both surfactant and G concentrations, albeit they do not depend on the G/surfactant weight ratio. For the same G concentration, the magnitude of the quenching follows the order: cationic > non-ionic ≥ anionic. The cationic surfactants, which strongly adsorb onto G via electrostatic attraction, are the most effective dispersing agents and they enable a stronger interaction with the zwitterionic form of the vitamin; the dispersing power improves with increasing the surfactant chain length. The fit of the experimental data to the Stern-Volmer equation suggests either a static or dynamic quenching mechanism for the dispersions in non-ionic surfactants, while those in ionic surfactants show a combined mechanism. The results that were obtained herein have been compared to those that were reported earlier for the quenching of another vitamin, riboflavin, to elucidate how the change in the vitamin structure influences the interactions with G in the surfactant dispersions.

## 1. Introduction

Graphene (G) is a very attractive nanomaterial with extraordinary electronic, magnetic, optical, and thermal properties [1], which is commonly used in a wide variety of applications ranging from electric/photonics circuits and solar cells [2], to polymer nanocomposites for medical [3] or high-performance uses [4]. Electronic excited states of molecules and chromophores can interact during their short lifetime with G, which influences their decay; this fluorescence quenching property of G has been used for the selective detection of biomolecules and other purposes [5]. Two main types of interaction have been described [6]: energy transfer and electron transfer. In the former, the singlet or triplet state of the chromophore relaxes back to the ground state transferring the energy to G. In the photoinduced electron transfer, the excited state accepts or gives an electron to G, thus creating a radical ion pair that matches with the state of charge separation. A critical issue is the quality of the nanomaterial, whether it is composed of single-layered, few-layered, or multilayer G (comprising more than 10 layers). The quenching effect of G has been described for a number of molecules, such as dyes [7,8], aromatic compounds [9,10], vitamins [11,12,13], and quantum dots [14]. Nonetheless, despite its huge potential applications, an in-depth analysis of the fluorescence quenching effect that was induced by G and the influencing factors, including molecular interactions and state of G dispersion, is missing in the literature.

Attaining stable dispersions of G in aqueous solutions is a major challenge that is owed to the attractive van der Waals forces among graphene flakes, which lead to the formation of irreversible aggregates. To solve this issue, different stabilization mechanisms that are used in colloidal science (i.e., electrostatic, steric, and electrosteric) have been implemented [15]. Further, G has been functionalized via covalent and non-covalent approaches [5]. The covalent functionalization includes amidation, esterification, and introduction of negatively charged carboxylic or sulfonic groups. The non-covalent method involves the use of surfactants and/or polymers that wrap around G to facilitate dispersion via hydrophobic, π–π, and ionic interactions [15,16].

Vitamin B_6_, also called pyridoxine, is an important biological molecule that plays a crucial role in protein metabolism [17]. It aids to keep the nervous system healthy, collaborates in the synthesis of neurotransmitters that influence mood, aids to make hemoglobin, to equilibrate blood sugar levels, and also to create antibodies [18]. The absence of pyridoxine can produce severe side effects, such as increased risk of heart and Alzheimer’s disease, rheumatoid arthritis, allergies, and other skin conditions, eye and bladder infections, etc. High intakes of vitamin B_6_ have been found to be significant at preventing cancer. Moreover, it has also been reported that the ingestion of vitamin B_6_ in cancer patients could improve their immune system [19]. When considering the high importance of vitamin B_6_ for human health, it is of great interest to develop analytical methods for the detection and quantification of this vitamin [20]. Among the developed procedures, one of the most common is fluorometry due to its simplicity, non-invasiveness, speed, outstanding sensitivity, selectivity and broad range of applicability. The sensitivity of the fluorescence technique can be further enhanced in organized micellar media, as demonstrated in our previous study [21].

**Scheme 1:**
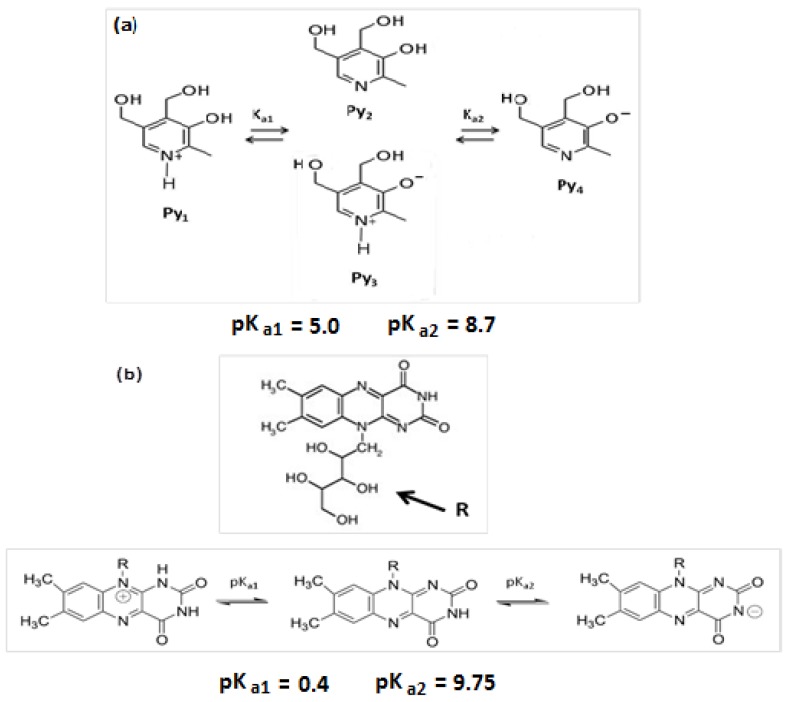
Structures of pyridoxine (**a**) and riboflavin (**b**) as a function of the solution pH.

Four possible structures for pyridoxine have been proposed [22] (Scheme 1a), and all of them are fluorescent. At pH < pKa_1_, the predominant species is the Py_1_ cation, which has a molecular absorption band at 290 nm. In the pKa_1_ < pH < pKa_2_ region, two possible structures can be found: the neutral species Py_2_ with an absorption band at 286 nm in the presence of alcohols, and the “zwitterionic” species Py_3_, in aqueous solutions, with a maximum absorption at 324 nm. At pH > pKa_2_, the predominant form is the Py_4_ anion with a maximum absorption at 310 nm. Consequently, it is essential to control the pH of the medium in the fluorescence experiments. 

**Scheme 2:**
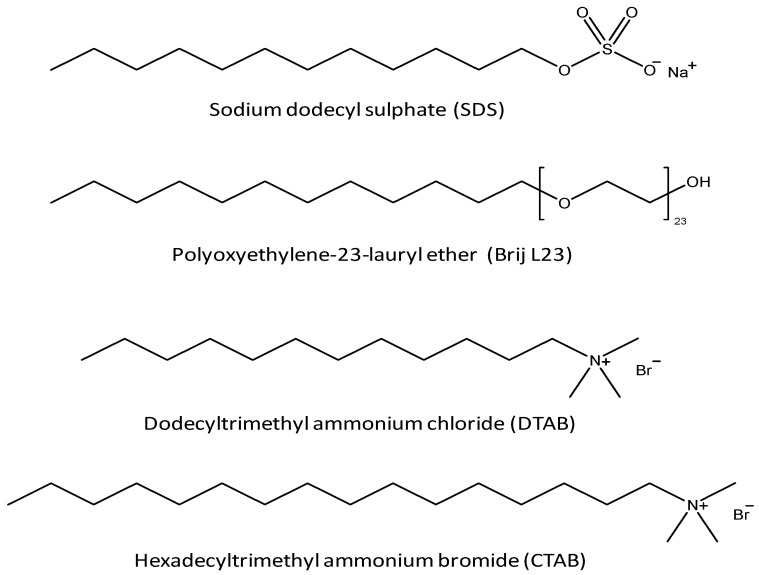
Structure of the surfactants used for the preparation of graphene (G) dispersions.

In a preceding work [23], G dispersions in aqueous solutions of surfactants of different nature: anionic sodium dodecylsulphate (SDS), non-ionic polyoxyethylene-23-lauryl ether (Brij L23), and cationic hexadecyltrimethyl ammonium bromide (CTAB) and dodecyltrimethyl ammonium chloride (DTAB) (Scheme 2) were prepared, and their influence on the fluorescence intensity of vitamin B_2_ (riboflavin) was examined. Riboflavin is a water soluble vitamin and it is considered as a polar molecule, despite that it comprises a hydrophobic aromatic moiety and a hydrophilic aliphatic chain with OH groups. Three structures can be described for riboflavin depending on the pH (Scheme 1b), and only the neutral form exhibits native fluorescence. The current study focuses on the effect of such G/surfactant dispersions on the fluorescence of pyridoxine, which is also a water soluble vitamin, albeit with a more polar structure (Scheme 1a). A systematic study has been carried out to gain information about the influence of the surfactant charge, chain length, and concentration, as well as the G/surfactant weight ratio and the G loading on the fluorescence intensity of pyridoxine. More importantly, the results have been compared with those that were obtained from the earlier work [23], in order to elucidate how the change in the vitamin structure influences on the interactions with G in the dispersions. The π–π stacking interactions between the aromatic rings of G and the pyridine ring of vitamin B_6_ or the aromatic isoalloxazine ring of vitamin B_2_ should be responsible for the quenching effect. Nonetheless, in the case of pyridoxine, the electrostatic attraction between the negatively charged electrons of the π system of G and the positive charge of the nitrogen of the pyridine ring can also play a key role in the quenching process. In addition, the interactions between the two vitamins and the different surfactant-wrapped G sheets have been examined herein. Thus, pyridoxine can strongly interact with the nanomaterial flakes that are coated by the ionic surfactants via electrostatic forces, whilst riboflavin, which is not charged at neutral pH, would mainly interact via weaker interactions, namely hydrogen bonding and polar forces between the OH groups of its ribityl side-chain and the polar moieties of the head group of the surfactants. The different nature and strength of the interactions will account for the differences observed. Further research in this direction would enable the development of G-based fluorescence sensors for the selective determination of pyridoxine or riboflavin in complex systems with a high degree of sensitivity.

## 2. Materials and Methods 

### 2.1. Reagents

Pristine graphene powder comprising more than 10 layers (see Raman spectrum, Figure S1 in the supplementary material), with lateral dimensions between 50 and 500 µm and an oxygen content of 3.5% (as determined by X-ray photoelectron spectroscopy (XPS)) [10] was provided by Avanzare Innovación Tecnológica, SL (Logroño, Spain). Pyridoxine (C_8_H_11_NO_3_, M_w_ = 169.18 g/mol) was provided by Calbiochem (Bogotá, Colombia). Sodium dodecylsulphate (SDS, NaC_12_H_25_SO_4_, micellar critical concentration CMC = 8.2 mM, M_w_ = 288.38 g mol^−1^), dodecyltrimethyl ammonium chloride (DTAB, CH_3_(CH_2_)_11_N(CH_3_)_3_Br, CMC = 14.0 mM, M_w_ = 308.34 g mol^−1^), and polyoxyethylene-23-lauryl ether (Brij L23, C_12_H_25_(OCH_2_CH_2_)_23_OH, CMC = 91 µM, M_w_ = 1198.56 g mol^−1^), were obtained from Sigma-Aldrich (Madrid, Spain). Hexadecyltrimethyl ammonium bromide (CTAB, C_19_H_42_BrN, M_w_ = 364.46 g mol^−1^, CMC = 0.9 mM) and KH_2_PO_4_ were purchased from Merck S.L.U. (Barcelona, Spain). K_2_HPO_4_ was obtained from D´Hemio Laboratories (Madrid, Spain). All of the solutions were prepared with ultrapure water from a Milli-Q system (Millipore, Milford, MA, USA).

### 2.2. Instrumentation 

Scanning electron microscopy (SEM) measurements at different magnifications were carried out with a Zeiss DSM-950 microscope (Carl Zeiss, Göttingen Germany) that was operating at 15 kV, under high vacuum. Upon drying for a few days, the G dispersions were covered with a ~10 nm Au layer in order to facilitate the visualization of the carbon nanomaterial. Thickness was estimated from the micrographs using ImageJ software (version 1.46c; WS Rasband, National Institutes of Health, Bethesda, MD, USA).

A Perkin-Elmer LS-50B spectrophotometer (Perkin-Elmer Instruments, Norwalk, CT, USA) was used to obtain the fluorescence spectra of G dispersions, as reported earlier [23,24]. Tip sonication was performed with a UP400S ultrasonic probe system (Hielscher Ultrasonics GmbH, Teltow, Germany), incorporating a titanium sonotrode of 7 mm diameter and approx 100 mm length.

### 2.3. Preparation of the Vitamin Solutions and the G Dispersions in the surfactants

A stock solution of pyridoxine of 200 mg L^−1^ was firstly prepared and stored under dark conditions. Working solutions of 40 mg L^−1^wereprepared by diluting the stock solution with water. The G dispersions (0.5, 1.0, and 2.0 wt %) in the different surfactants were prepared following a similar procedure to that reported previously [23]. The following surfactant concentrations were selected, all of them being significantly above the CMC: 10 mM Brij L23, 20 mM SDS, and CTAB, as well as 30 mM DTAB. Briefly, the necessary amount of each component was weighed and diluted with ultrapure water. The solutions were bath sonicated for 1 h and were subsequently tip sonicated for 5 min at 160 W and 24 kHz. Then, the dispersions were centrifuged at 2147 g for 1 h and were kept in amber containers. In the case of Brij L23, solutions were prepared in phosphate buffer (pH = 7). To ensure repeatability, all of the dispersions were prepared at least twice.

### 2.4. Fluorescence Spectra of Pyridoxine in Surfactant Solutions and G Dispersions in the Surfactants

The fluorescence spectra were acquired at T = 25 ± 0.1 °C and pH = 7.0, as reported elsewhere [13,23]. The first excitation wavelength (λ_exc_) was set at 230 nm, and 15 spectra were recorded with Δλ of 10 nm to determine the best λ_exc_ and λ_em_ (emission wavelength), as well as the corresponding fluorescence intensities. For each G percentage studied, two series of dispersions were prepared: one by diluting the initial G dispersion in the surfactant with water, in order to attain different G and surfactant concentrations whilst maintaining the G/surfactant weight ratio constant, and the other by dilution with the initial surfactant concentration in order to keep it constant while changing the G concentration and the G/surfactant weight ratio. Subsequently, 200 µL of the vitamin solution was added to each of the dispersions, thus the final concentration of the vitamin for the fluorescence measurements was 0.8 mg L^−1^.

## 3. Results and Discussion

### 3.1. Assessment of the Quality of G Dispersions in the Different Surfactants

The quality of G dispersions was evaluated via SEM. The average flake thickness gives information about the level of G exfoliation, which is a factor that has a noticeable influence on the quenching effect, as will be shown in the following sections. Figure S2 in the supplementary material shows images of pristine G at different magnifications. The raw nanomaterial comprises a number of stacked G sheets that are bounded by π–π stacking interactions and van der Waals forces. The observed flakes are relatively thick and stiff, with thicknesses that are in the range of 78–30 nm and an average value of 58 ± 11 nm. Nonetheless, due to the high error in the measurement method (≥10%), and when considering that while sample drying stacking can occur to minimize the surface energy, the values that are obtained herein should be taken as an approximation and for comparative purposes only.

To gain more insight into the graphene layer stacking, the Raman spectrum of pristine G was recorded (Figure S1). According to the work by Hao et al. [25], the position and width of the 2D peak provides information about the number of G layers: the broader the band and the higher its frequency, the larger the number of G flakes. In particular, the full width at half maximum (FWHM) of the 2D band has been reported to be about 27.5, 51.7, 56.2, 63.1, and 66.1 cm^−1^ for single-, bi-, tri-, four-, and five-layer graphene [25]. For G that is thicker than five layers, the Raman spectrum is hardly distinguishable from that of bulk graphite. The graphene that was used in the current study has a 2D band with a FWHM value of 93 cm^−1^, which strongly suggests that comprises more than five layers. Further, the 2D band appears close to 2700 cm^−1^, which is also characteristic of G thicker than five layers [25,26]. Therefore, the combination of Raman and SEM suggests that the pristine nanomaterial indeed corresponds to multilayer graphene, with more than 10 layers.

Regarding G (0.5 wt %) dispersions in 20 mM SDS (Figure S3 in the supplementary material), a noticeable fall in the flake thickness is found, with the average thickness being 35 nm. This indicates that SDS acts as dispersant agent of G, thus promoting the exfoliation of the nanomaterial into thinner flakes. Besides, the sheets are randomly and uniformly dispersed in the presence of the surfactant, and they look more flexible than those of pristine G. It is important to note that the exfoliation attained herein is not complete, since no individual flakes can be observed in the micrographs. This is in contrast to the results that were reported previously for G dispersions in another anionic surfactant, sodium cholate (SC) [27], where monolayers with a width of ~1 µm were attained after low power sonication up to 400 h. The longer sonication times and the planar π–π structures of SC that enable stronger interaction with G than the short hydrocarbon chains of SDS result in a better G dispersion. In the dispersions with 1.0 and 2.0 wt % G in SDS (Figure S3), the level of exfoliation that is attained seems poorer, with the mean thicknesses being 42 and 46 nm, respectively, and the sheets exhibit lower flexibility.

The images of G dispersions in 10 mM Brij L23 (Figure S4 in the supplementary material) reveal that G is quite well exfoliated and dispersed for the three percentages studied, although when the G/surfactant weight ratio is very low, the nanomaterial sheets present a soapy appearance, indicating that they are highly coated by Brij L23, and appear thicker (Figure S4a–c). Accordingly, the average flake thickness is 41, 36 and 28 nm for dispersions with 0.5, 1, and 2 wt % G, respectively. This confirms that a small amount of Brij L23 is enough to attain a good G dispersion.

The micrographs of the dispersions in DTAB (Figure S5 in the supplementary material) show in all cases a uniform and random distribution, with very flexible sheets that are much more separated than in the raw G. For the same G percentage, the degree of exfoliation achieved is greater than that of the dispersions in Brij L23 and in SDS. In addition, unlike SDS, the level of exfoliation is maintained or even slightly improved upon increasing the G/surfactant weight ratio, with average flake thickness of 21, 23, and 15 nm for G percentages of 0.5, 1, and 2 wt %, respectively. These results suggest that DTAB is a very effective dispersing agent since it is able to exfoliate and disperse very well all of the G concentrations studied in this work.

With regard to G dispersions in CTAB (Figure S6 in the supplementary material), a very homogeneous distribution is observed with well exfoliated and highly flexible sheets. The degree of exfoliation obtained is significantly greater than that found for the dispersions in the anionic and nonionic surfactants, and is slightly better than that achieved with the DTAB, with an average flake thickness of 19, 16, and 12 nm for G percentages of 0.5, 1, and 2 wt %, respectively, indicating that the nature of the surfactant has greater influence than its chain length on the level of dispersion that is reached. In addition, similarly to the dispersions in DTAB, the degree of exfoliation improves slightly with increasing the G/surfactant weight ratio.

### 3.2. Fluorescence of Pyridoxine in Aqueous Surfactant Solutions

**Figure 1:**
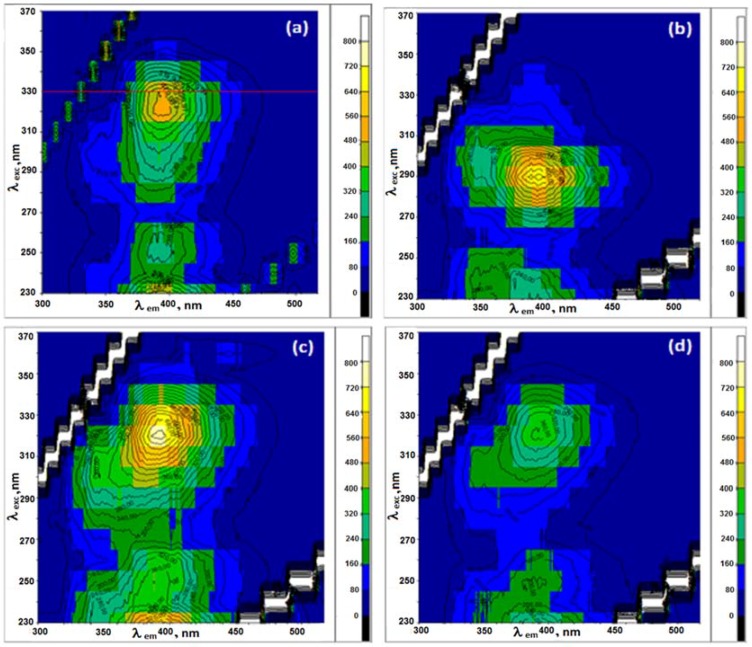
Three-dimensional (3D) fluorescence contour maps of (**a**) pyridoxine (0.8 mg L^−1^) in aqueous solution (pH = 5.9), (**b**) 10 mM Brij L23 in aqueous solution (pH = 3.4), (**c**) 10 mM Brij L23 in phosphate buffer (pH = 7.0), and (**d**) 1 wt % G dispersion in 10 mM Brij L23 in buffer (pH = 7.0).

The three-dimensional (3D) fluorescence contour map of pyridoxine in aqueous solution (pH = 5.9) is shown in Figure 1a. The analysis of these spectra provides relevant information, since it allows for selecting the experimental measurement conditions. The wavelength of maximum emission intensity (λ_em_) is 395 nm, corresponding to a λ_exc_ of 325 nm. This λ_em_ was selected to explore the effect of aqueous solutions of the different surfactants studied, at concentrations above and below their CMC, on the fluorescence intensity of vitamin B_6_. In the case of Brij L23 solutions (pH = 3.4), a hipsocromic shift of λ_exc_ from 328 to 290 nm was found (Figure 1b), which is likely related to the change in the solution pH. Thus, as mentioned in the introduction, at pH < 5, the predominant species is the Py_1_ cation that shows the maximum of the absorption band at 290 nm (Scheme 1). This is in contrast to the results that were found for riboflavin in the presence of the same surfactants, where no change in λ_exc_ nor in λ_em_ was detected, indicating that the polarity surrounding the vitamin was not modified and that riboflavin does not solubilize in the micelles.

**Figure 2:**
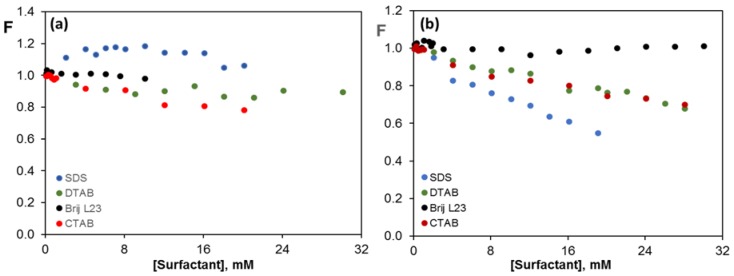
Change in the fluorescence (F) of vitamin B_6_ (λ_exc_ = 325 nm; λ_em_ = 395 nm) (**a**) and vitamin B_2_ (λ_exc_ = 450 nm; λ_em_ = 518 nm) (**b**) with increasing concentration of the different surfactants.

With the aim to compare the results that were obtained for all the surfactants, Brij L23 solutions were prepared in this work in phosphate buffer (pH = 7, Figure 1c), so that the predominant form was the zwitterionic Py_3_. The change in the maximum fluorescence intensity (F) of pyridoxine normalized to the value of the vitamin in water as a function of the surfactant concentration is shown in Figure 2a.

In the case of SDS solutions (pH = 7.4), a slight increase in F is found at very low surfactant concentrations (≤2 mM), while at higher concentrations it remains constant, indicating that there is no quenching effect that is caused by this surfactant on the fluorescence intensity in the absence of G. At neutral pH, the predominant form of vitamin B_6_ is the “zwitterionic” Py_3_ (Scheme 2). The small rise in intensity that was found at low SDS concentrations is likely due to a change in the effective dielectric constant of the environment surrounding the vitamin, hence the micro-polarity, upon the addition of the surfactant. This is consistent with previous works that studied the variation of microenvironmental parameters of fluorescent probes in the presence of SDS solutions, and found a gradual change in the micropolarity with an increasing surfactant concentration [28]. Besides, the increase in intensity can also be induced by the interactions between SDS monomers and vitamin B_6_ via formation of an ionic par between the negative charge of SDS monomers and the positive charge of the nitrogen of the pyridine ring. The fact that F remains approximately constant above and below the CMC suggests that the vitamin does not situate around nor penetrate the SDS micelles. Due to its high polarity, it likely prefers to be located in the bulk solution (highly polar medium) instead of on the micelles surface (medium polar environment) or inside the micelle (non-polar medium). The aforementioned behaviour is in contrast to that reported for the fluorescence intensity of riboflavin in the presence of SDS solutions (Figure 2b, reprinted from [23] with permission from MDPI), where a significant drop in intensity was found with increasing surfactant concentrations, which is indicative of a strong interaction between riboflavin and the surfactant molecules. Since riboflavin is less polar than pyridoxine, it would be more prone to penetrate inside the SDS micelles, which results in a decrease in the fluorescence intensity.

On the other hand, no quenching effect of pyridoxine fluorescence is observed in the presence of Brij L23 solutions (Figure 2a). Since this surfactant does not have charged functional groups, it would weakly interact with vitamin B_6_ via hydrophobic interactions, van der Waals forces, H-bonding, and polar interactions between its ether groups and the nitrogen and OH groups of the vitamin. Given that vitamin B_6_ is highly soluble in water and it weakly interacts with this non-ionic surfactant, its solubility hardly changes; hence, F remains almost constant with an increasing Brij L23 concentration. Very similar behaviour was found for the fluorescence of riboflavin in the presence of this surfactant (Figure 2b) [23], or in aqueous solutions of hydrophilic polymers, like polyethylene glycol (PEG) [11], and triblock copolymers, like polaxamer 407 [13], ascribed to the weak interactions between riboflavin and the polymers that did not alter the vitamin fluorescence intensity.

Experimental results reveal hardly changes in F of pyridoxine in the presence of the cationic surfactants (Figure 2a), also indicative of weak vitamin-surfactant interactions, which could be related to the electrostatic repulsion between the positive charge of the nitrogen of the pyridine ring and that of the surfactants. Hence, the vitamin likely prefers to locate in the bulk solution, far from the micellar environment. In contrast, a fall in the fluorescence of riboflavin has been detected in the presence of both cationic surfactants (Figure 2b) [23], hinting towards a stronger vitamin-surfactant interaction, since riboflavin has no charge at neutral pH. Further, CTAB and DTAB, which only differ in the chain length (Scheme 2), exerted the same quenching effect, indicating that the surfactant charge is the crucial factor controlling the quenching behaviour.

### 3.3. Fluorescence of Pyridoxine in the Presence of G Dispersions in the Different Surfactants

Dispersions with G/surfactant weight ratios of 0.5, 1.0, and 2.0% were prepared in the four surfactants that were investigated, and two series of measurements were performed: one by simultaneously changing the concentration of the surfactant and G while maintaining the G/surfactant weight ratio (w_G_/w_S_) constant, and the other by varying the G concentration and the G/surfactant weight ratio, whilst keeping the surfactant concentration unaltered.

#### 3.3.1. Graphene Dispersions in 20 mM SDS Aqueous Solutions

**Figure 3:**
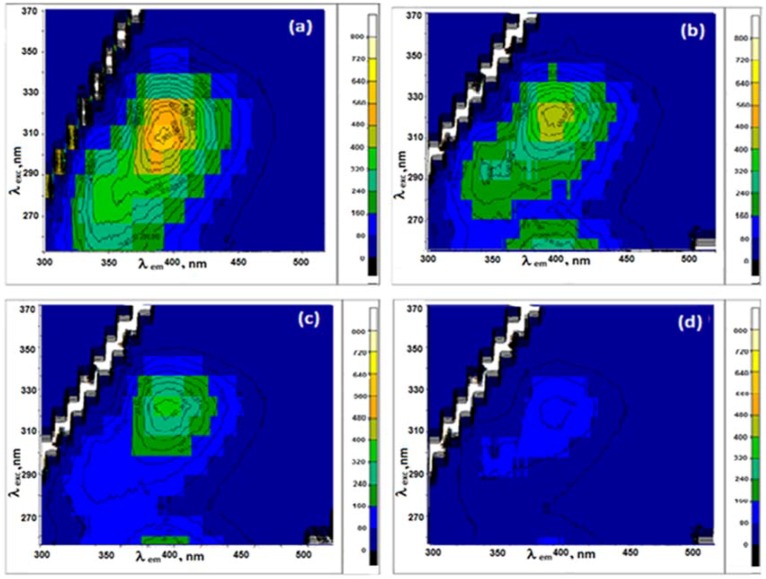
3D fluorescence contour maps of pyridoxine in (**a**) 20 mM SDS and G (0.5, 1.0, and 2.0 wt %) dispersions in 20 mM SDS (**b**, **c**, and **d**, respectively).

**Figure 4:**
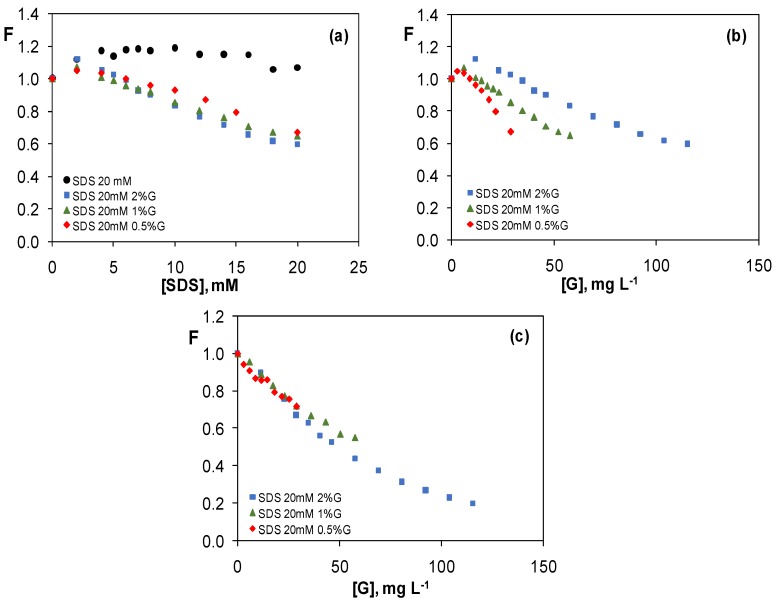
Evolution of the fluorescence of vitamin B_6_ (λ_exc_ = 325 nm; λ_em_ = 395 nm) in the presence of G dispersions in 20 mM SDS vs. surfactant (**a**) and graphene (**b**,**c**) concentration for solutions with a constant G/SDS weight ratio (**a**,**b**) or constant SDS concentration (**c**).

Figure 3 shows the 3D contour maps of pyridoxine in the presence of 20 mM SDS aqueous solution and G dispersions (0.5, 1.0, and 2.0 wt %) in 20 mM SDS. The change in F as a function of SDS (a) and G (b,c) concentration for solutions with a constant w_G_/w_S_ (a,b) or constant SDS concentration (c) is illustrated in Figure 4. It is important to note that in the case of dispersions prepared by dilution with water, the fluorescence intensity was normalized to the value of the vitamin in water, whilst for dispersions that were prepared by dilution with the initial surfactant concentration it was normalized to the intensity value in such surfactant concentration (i.e., 20 mM for SDS solutions).

A clear drop in F is found with an increasing SDS concentration (Figure 4a), indicating a quenching phenomenon that is provoked by the presence of G in the surfactant solutions. These results are consistent with SEM observations (Figure S3), which revealed improved G dispersion with an increasing surfactant weight ratio. Taking into account that the same reduction in F, which is close to 40%, is attained for G concentrations of 30, 60, and 120 mg L^−1^ (Figure 4b), it can be concluded that the quenching in this system does not depend on the total G concentration that is added. Further, for the same surfactant concentration, the quenching hardly changes with increasing the G/surfactant weight ratio (Figure 4c), hinting that the amount of G that is dispersed in the medium is the same for the three percentages studied.

When considering that SDS in the absence of G slightly changes the fluorescence intensity (Figure 2a), it appears that the quenching is caused by the simultaneous presence of G and SDS, albeit it does not depend on their relative proportions but on the total SDS concentration. This fact hints that the G sheets that are wrapped by the surfactant interact more strongly with pyridoxine than the uncovered nanomaterial flakes that are free in the solution. Given that the drop in intensity is gradual, and it takes place at surfactant concentrations both below and above the CMC (Figure 4a), it can be inferred that the pyridoxine-monomer and pyridoxine-micelle interactions are similar, and that the surfactant monomers are mainly responsible for the nanomaterial dispersion and hence the observed quenching effect. As the SDS concentration increases, the number of SDS monomers that are adsorbed onto G should increase, hence the greater would be the number of negative charges, and the stronger should be the interaction with pyridoxine. Future work will determine the amount of surfactant absorbed per G flake to corroborate this hypothesis. According to literature [29], SDS monomers adsorb onto G with their hydrocarbonated chains being oriented parallel to the graphitic basal plane, forming a monolayer where the surfactant-rich and surfactant-poor region coexist. Thus, G flakes that are covered by SDS have a negative charge and are stabilized versus re-aggregation that is owed to repulsive electrostatic interactions between adjacent layers.

The abovementioned results differ from those that were previously reported for the fluorescence of riboflavin [23], where the quenching effect became stronger with increasing w_G_/w_S_ as well as both G and SDS concentrations. Besides, the plot of the fluorescence data as a function of SDS concentration showed a change in slope in the vicinity of the CMC, suggesting different interactions of the vitamin with the SDS monomers and micelles. Further, for the same G total concentration (i.e., 120 mg L^−1^) and the same G/surfactant weight ratio (i.e., 2%), the quenching effect is higher for vitamin B_6_ (about 80%) when compared to vitamin B_2_ (around 63%). The different behavior should be related to different interactions between the vitamin and the SDS-wrapped G flakes. Thus, pyridoxine can strongly interact with the surfactant-coated sheets via electrostatic attraction between the positively charged nitrogen of the pyridine ring and the negatively charged polar heads of SDS, as well as through “catión-π” interactions between the positive charge of the nitrogen and the π cloud of G. Further, hydrogen bonds can be formed between the OH groups of the vitamin and the π orbitals of G that can act as proton acceptors as well as with the oxygen atoms of the SDS head, and C–H⋯π interactions can occur between the C–H groups of the aliphatic SDS chain and the aromatic pyridine ring [30]. However, since riboflavin is not charged at neutral pH, then it would interact with the SDS-coated sheets via weaker interactions, namely hydrogen bonding and polar interactions between its OH groups and the polar moieties of the SDS head, C–H⋯π interactions between the aliphatic SDS chain and its aromatic rings, π–π stacking interactions with the aromatic rings of G and van der Walls forces. Thus, the weaker interactions would be responsible for a less pronounced quenching for a given G concentration. Overall, the results hint that in the case of riboflavin, the π–π interactions between SDS-wrapped G and the vitamin predominate, whereas for pyridoxine, the most important are the electrostatic attractions between G that is covered by the SDS monomers and the zwitterionic form of vitamin B_6_. Nonetheless, for both vitamins, a partition equilibrium between the surfactant-coated flakes, the SDS micelles in the bulk solution, and the free G sheets in solution would occur.

#### 3.3.2. Graphene Dispersions in 10 mM Brij L23 Aqueous Solutions

**Figure 5:**
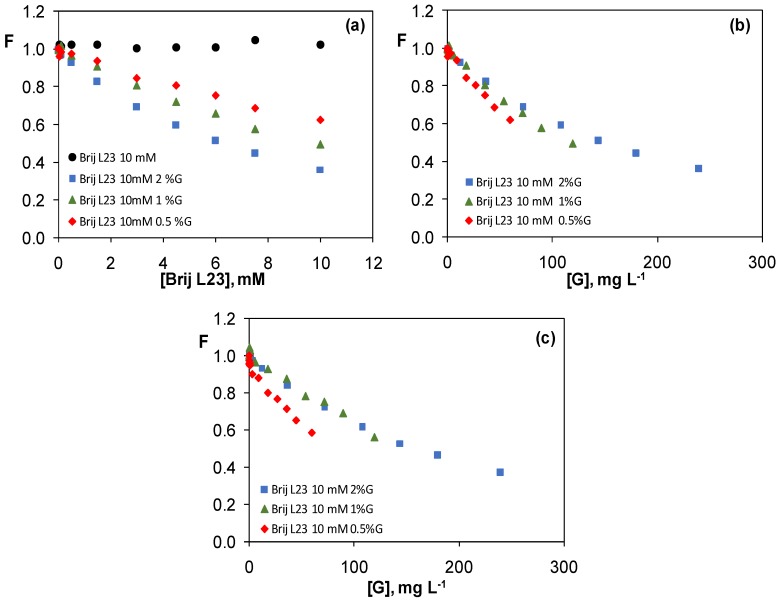
Change in the fluorescence of pyridoxine (λ_exc_ = 325 nm; λ_em_ = 395 nm) in the presence of G dispersions in 10 mM Brij L23 vs. surfactant (**a**) and graphene (**b**,**c**) concentration for solutions with a constant w_G_/w_S_ (**a**,**b**) or a constant Brij L23 concentration (**c**).

Figure 5 shows the change in the fluorescence of vitamin B_6_ in the presence of G dispersions in 10 mM Brij L23 vs. surfactant concentration (a) or G concentration (b,c) for solutions with a constant w_G_/w_S_ (a,b) or a constant Brij L23 concentration (c). In all cases, a decrease in F is found, which is indicative of a quenching effect that becomes more pronounced with increasing both G and Brij L23 total concentration. Nonetheless, when considering that a higher surfactant concentration is linked to an increase in the G content, it seems that the trend observed is due to the higher nanomaterial concentration. Thus, the higher the total G content, the higher the amount of G that is dispersed in the medium available to interact with the vitamin, which is reflected in more effective quenching. In general, at very low Brij L23 concentrations, below the CMC, F hardly changes, whilst it decreases significantly above the CMC, suggesting that the quenching is more effective at concentrations > CMC, when micelles are formed. Thus, it appears that the G flakes that are wrapped by the micellar aggregates interact more strongly with G than the monomers. This behaviour is opposite to that found for G dispersions in SDS, where the monomers were mainly responsible for the quenching effect.

On the other hand, very small differences are found between the three G percentages that are studied (Figure 5b,c), which is in agreement with SEM observations (Figure S4) that revealed a similar exfoliation degree for the three G/surfactant weight ratios. In this case, Brij L23 has a main role as dispersing agent of the G flakes; given that it is a non-ionic surfactant, it would only interact weakly with pyridoxine via H-bonding and polar interactions, as mentioned earlier. Therefore, the π–π interactions between the aromatic ring of G and the pyridine ring of vitamin B_6_ should be responsible for the quenching, hence it would mostly occur with the G flakes that are slightly covered by the surfactant.

Very similar conclusions were drawn for the quenching of riboflavin fluorescence by G dispersions in Brij L23 [23], where the three G percentages almost led to the same effect, hinting that the quenching depended mostly on the G amount in the solution. The non-ionic surfactant wraps around the G flakes, and it is the uncovered G surface that mainly interacts with the vitamin. Similar to pyridoxine, the key mechanism of interaction is the π–π stacking between the electron-donor isoalloxazine rings of vitamin B_2_ and the electron-acceptor aromatic rings of the G dispersed in the solution. Hence, for both vitamins, a maximum F reduction of about 60% is found for a G concentration of 240 mg L^−1^ and a G/Brij L23 weight ratio of 2%.

The stabilization mechanism of G flakes in aqueous solutions of non-ionic surfactants, like Brij L23, involves different interactions, such as polar, hydrophobic, steric, and van der Waals [12]. Thus, the hydrocarbonated chain of the surfactant adsorbs onto the G sheets by means of van der Waals and hydrophobic interactions, whereas the bulky hydrophilic groups extend into the water. When two Brij-wrapped flakes approach, the hydrophilic groups interact, thus provoking repulsion between the flakes.

#### 3.3.3. Graphene Dispersions in 30 mM CTAB and 20 mM DTAB Solutions

**Figure 6:**
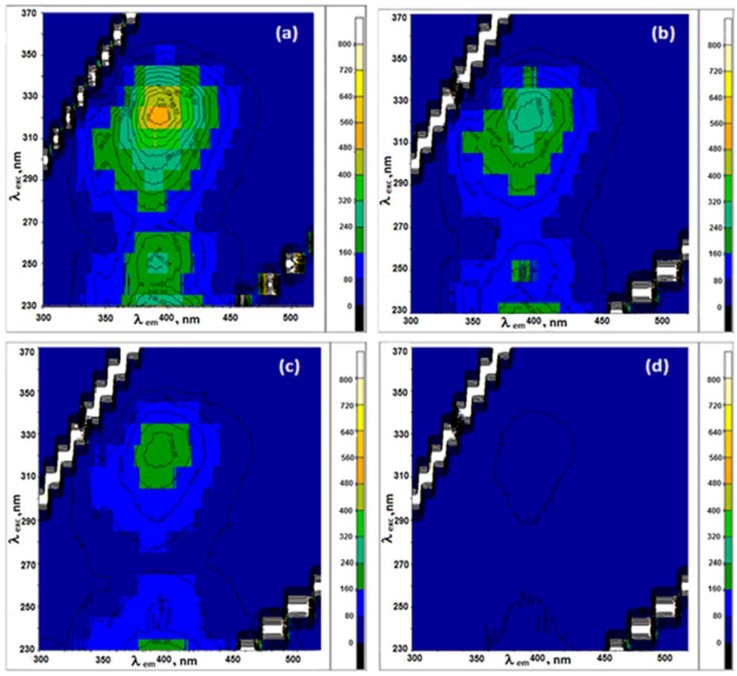
3D contour maps of pyridoxine in (**a**) 20 mM CTAB and G dispersions in 20 mM CTAB: 0.5 wt % (**b**), 1 wt % (**c**), and 2 wt % (**d**).

Two cationic surfactants, DTAB and CTAB, with the same trimethylammonium head group, albeit with different hydrocarbonated chain length (twelve and sixteen carbon atoms, respectively), were selected to investigate the effect of the chain length on the fluorescence of pyridoxine. Given that the pH of the DTAB and CTAB aqueous solutions was 5.5, the predominant species of the vitamin was the zwitterionic Py_3_, hence no buffer was used_._ Figure 6 displays the 3D spectra of vitamin B_6_ in 20 mM CTAB, as well as in G dispersions (0.5, 1 and 2 wt %), in the surfactant solution.

**Figure 7:**
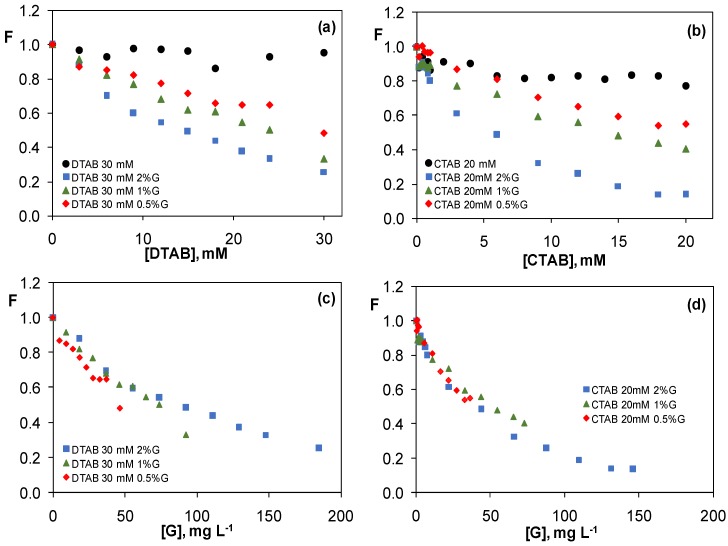
F (λ_exc_ = 325 nm; λ_em_ = 395 nm) vs. surfactant (**a**,**b**) or graphene (**c**,**d**) concentration for G dispersions in 30 mM DTAB (**a**,**c**) and 20 mM CTAB (**b**,**d**) with a constant G/surfactant weight ratio.

The fluorescence of vitamin B_6_ vs. surfactant (a,b) or G (c,d) concentration for G dispersions in 30 mM DTAB (a,c) and 20 mM CTAB (b,d) with a constant w_G_/w_S_ is shown in Figure 7. The comparison of Figure 7a,b indicates that while in the absence of G, the trend for both of the surfactants is very similar, in the presence of G the behaviour somewhat differs, and CTAB with a longer chain leads to a stronger quenching. This is in agreement with SEM observations, which revealed better degree of G exfoliation for this surfactant when compared to DTAB, hence more surface area is available to interact with pyridoxine. For both DTAB and CTAB, F decreases with an increasing G and surfactant concentration, leading to reductions of about 65 and 90%, respectively, for a G concentration of 150 mg L^−1^ and a w_G_/w_S_ of 2%.

Note that the quenching efficiency that was observed for CTAB is very significant; such a strong quenching effect in the presence of G has only been described for proteins incorporating aromatic rings [31]. The very intense quenching that is found for CTAB is likely due to a synergic effect of a high amount of G dispersed and a high concentration of micelles, since the surfactant concentration is about 20 times higher than the CMC, hence a great number of positive charges that are suitable to interact with the vitamin. Thus, the positive charges of the polar heads of DTAB and CTAB can strongly interact via electrostatic attraction with the negatively charged oxygen of pyridoxine in the zwitterionic form (Scheme 1). Further, hydrogen bonds can be formed between the nitrogen of the trimethylammonium head and the OH groups of pyridoxine, as well as C–H⋯π interactions between the C–H of the aliphatic chain of the cationic surfactants and the aromatic pyridine ring.

For both of the surfactants, the drop in F hardly changes with increasing the G/surfactant weight ratio, which is consistent with SEM images (Figures S3 and S4) that revealed a quite similar level of G dispersion for the three G percentages. In the case of DTAB (Figure 7c), the drop is slightly more pronounced for the dispersion with 0.5 wt %, indicating that the G flakes that are well covered by the surfactant interact more strongly with the vitamin.

The aforementioned results considerably differ from those that were previously reported for the fluorescence of riboflavin [23]; in such case, G dispersions in DTAB, with a smaller hydrocarbon chain, led to a stronger quenching. Unexpectedly, for dispersions in this surfactant, the three G percentages that were studied exerted the same effect on the fluorescence of riboflavin, whereas for dispersions in CTAB, the greater the amount of G, the more pronounced the quenching effect. This unexpected behaviour was explained [24] when considering that a greater number of DTAB monomers with shorter chain than CTAB can adsorb onto the G flakes. In addition, taking into account that the CMC of DTAB is 15.5 times larger than the CMC of CTAB, the concentration of monomers in the former dispersions is higher, hence resulting in a stronger quenching effect. The opposite behaviour found when compared to pyridoxine can be understood while considering that its predominant form at neutral pH is the zwitterionic, hence the interaction with the cationic surfactants is electrostatically driven, as explained above. Therefore, the greater the number of charges, the stronger the interaction. However, since riboflavin is not charged, it would mainly interact with the surfactant-wrapped sheets via hydrogen bonding and polar interactions between its nitrogen and OH groups, and the trimethylammonium polar head of CTAB or DTAB. Besides, C–H⋯π interactions can occur between the aliphatic chain of the cationic surfactants and its aromatic rings, as well as π–π stacking interactions with the aromatic rings of G. Consequently, the greater the number of monomers, the more efficient the quenching is.

**Figure 8:**
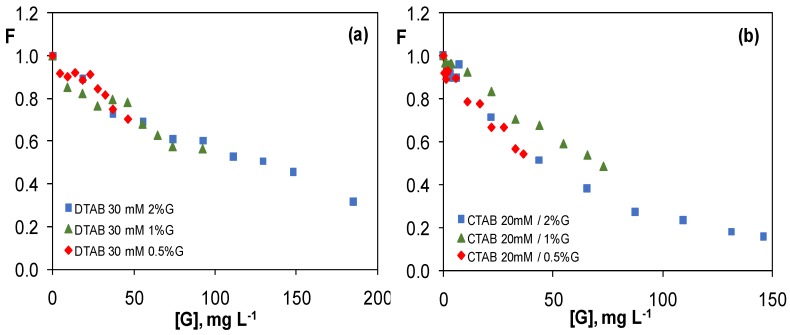
F (λ_exc_ = 325 nm; λ_em_ = 395 nm) vs. G concentration for G dispersions with a constant surfactant concentration: 30 mM DTAB (**a**) and 20 mM CTAB (**b**).

Figure 8 shows the change in the fluorescence of pyridoxine with G concentration for dispersions with a constant surfactant concentration: 30 mM DTAB (a) and 20 mM CTAB (b). Again, the magnitude of the quenching hardly changes with increasing the G/surfactant weight ratio, and in the case of CTAB, it is somewhat more pronounced for the dispersion with 2% G when compared to that with 1%, which could be related to the slightly better level of G exfoliation, according to SEM images (Figure S6).

According to previous works [32], the mechanism of stabilization of G dispersions in cationic surfactants is similar to that in anionic ones. The hydrocarbonated surfactant chains adsorb onto the G surface via hydrophobic interactions and the polar heads extend into the aqueous medium and impart an effective positive charge to the sheet. Thus, the electrostatic repulsions between adjacent flakes stabilize the dispersion. However, a new model that is based on electrostatic forces has been recently proposed [33] in order to explain the interaction and structure of the complexes formed between CTAB and graphene oxide (GO), the oxidized form of G. It consists in an alternating multilayer assembly where CTAB bilayers intercalate between GO layers. Thus, the positively charged heads of the cationic surfactant would electrostatically interact with the negatively charged groups (i.e., O–, CO– and COO–) of the GO surface. Given that the graphene that was used in this study has a few oxygen-bearing functional groups (~3.5 wt %, as determined by XPS) [10], it is more similar to reduced graphene oxide (rGO) than to hydrophobic pristine graphene. Therefore, it is reasonable to assume that a similar intercalated assembly to that reported in [33] could be formed between DTAB or CTAB and the graphene employed herein, with the surfactant micelles interacting with the oxygen moieties of the G surface, hence leading to a very effective dispersion of the G sheets in aqueous medium.

### 3.4. Comparison of the Quenching Effect of Graphene Dispersions in the Four Surfactants

**Figure 9:**
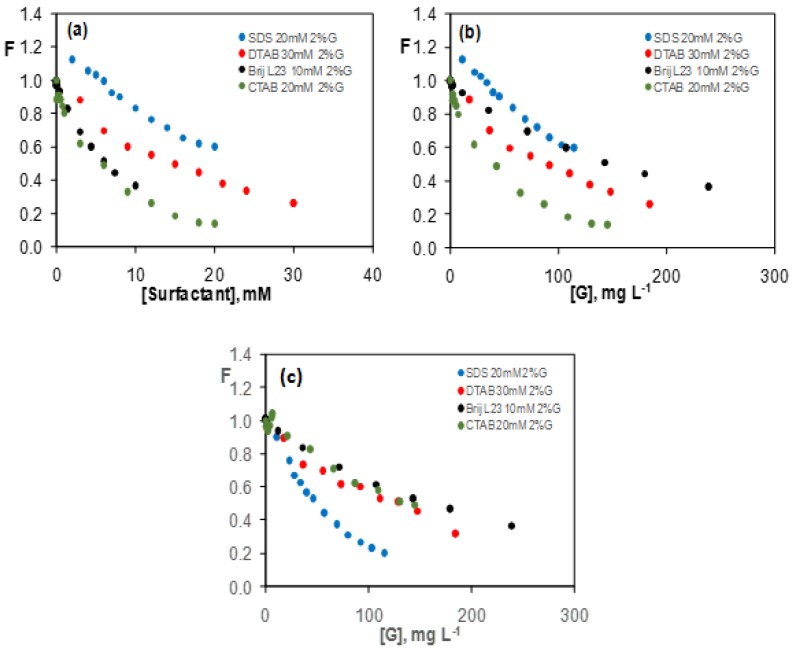
Comparison of the quenching effect (λ_exc_ = 325 nm; λ_em_ = 395 nm) for G (2 wt %) dispersions in the indicated surfactant solutions vs. surfactant (**a**) or G (**b**,**c**) concentration, for dispersions with a constant w_G_/w_S_ (**a**,**b**) or a constant surfactant concentration (**c**).

Figure 9 compares the evolution of F as a function of the surfactant (a) and G (b) concentration for G (2 wt %) dispersions in the four surfactants that were investigated, keeping w_G_/w_S_ constant. For each surfactant, data were normalized to the value of the fluorescence intensity of pyridoxine in the absence of G. The reduction in F induced by the G dispersions in all of the surfactants exhibits a similar trend, with a stronger drop at low surfactant (Figure 9a) and G (Figure 9b) loadings. For the same G concentration, the magnitude of the quenching follows the order: CTAB > DTAB > Brij ≥ SDS. For instance, for a G concentration of ~115 mg L^−1^, the quenching for CTAB dispersion is about 3.2 times higher than that of Brij or SDS dispersions, and around 2.3-fold that of DTAB dispersion. These results are in very good agreement with the flake thickness that was obtained from SEM images (Figures S3–S6). Thus, CTAB dispersions exhibit the finer sheets, followed by DTAB ones, while SDS dispersions present thicker and poorly dispersed flakes. Interestingly, a 10-fold fall in the fluorescence of pyridoxine is attained for the dispersion in CTAB when a G concentration of 150 mg L^−1^ is reached. This drop is higher than that described for the quenching effect of G on the fluorescence intensity of substances comprising aromatic rings, such as phthalocyanines [34] or dyes [35], accounting for the high effectiveness of this surfactant as a dispersing agent of G sheets in aqueous solutions.

Experimental results confirm that the magnitude of the quenching effect depends strongly on the nature of the surfactant and only slightly on its chain length, hence on the size of the micelles, and that the cationic surfactants are the most suitable for exfoliating G in aqueous solutions. This is consistent with the results from the previous study [24], which demonstrated that G dispersions in the cationic surfactants were the most efficient in decreasing the fluorescence intensity of riboflavin. Further, it is also in good agreement with former works [25] that proved that non-ionic surfactants are less efficient than ionic ones in dispersing G, which suggests that strong G-surfactant interactions are needed to achieve a good G exfoliation, as well as stable and homogenous aqueous dispersions. Besides, for surfactants of the same nature, the longer the chain length, the more pronounced the quenching effect, given that the intensity of the van der Walls forces increases with increasing the chain size. Thus, longer molecules can adhere more strongly to G and lead to better dispersions. This could also explain the fact that the quenching is somewhat stronger for G dispersions in CTAB when compared to those in DTAB.

The structure of the surfactant chain adsorbed onto G is another factor than influences on the dispersion. Thus, it is believed that SDS adsorbs onto G via the formation of a monolayer or hemicilindrical micelles that do not completely cover the G Surface [36], which is reflected in a low surface charge density. Further, it appears to be a direct correlation between the surface charge density, which rises with increasing the chain length up C_16_ [37], and the effectiveness of the surfactant as dispersing agent. The fact that the magnitude of the quenching in the presence of G dispersions in Brij L23, which is a non-ionic surfactant, is similar to that of the dispersions in SDS is likely related to its higher molecular weight (about four times that of SDS) and the great steric hindrance of its bulky (O–CH_2_–CH_2_) groups that impede the re-aggregation of the G sheets [16].Overall, ionic and non-ionic surfactants display different stabilization mechanisms of G aqueous dispersions that can be explained by the colloidal stability theory. Results confirm that cationic surfactants, which strongly adsorb onto G via electrostatic attraction, are the most effective in exfoliating G in aqueous solution, especially those with high molecular weight. This is consistent with previous studies that compared the dispersion capability of surfactants of different nature and molecular weight [38].

### 3.5. Study of the Quenching Phenomenon

Any phenomenon that reduces the fluorescence intensity of a fluorophore is known as quenching, and it can be the outcome of several processes, namely energy transfer, complex formation, excited state reactions, and collisions [6]. There are two models by which quenching is usually described: the quenching arising from collisions between the quenching agents and fluorophores is called collisional or dynamic quenching. This kind of quenching is ruled by the diffusion speed of the quencher throughout the solution to collide with the fluorophore, and the fluorescence intensity depends on the quencher concentration [Q], according to the expression:

F_0_/F = 1 + k_q_τ_0_[Q] = 1 + K_SV_[Q] (1)

where F_0_ and F are the fluorescence intensities without and with quencher, K_SV_ is the Stern−Volmer constant, k_q_ is the rate constant of the quenching process, and τ_0_ is the lifetime of the fluorescent state in the absence of quencher.

When the quenching agent forms a non-fluorescent complex with the fluorophore, typically via formation of a ground-state complex, it is named as static quenching, and can be described by the equation:

F_0_/F = 1 + K_S_[Q] (2)

where K_S_ is the binding constant between the fluorophore and the quencher. Deviations from the linearity can take place as a result of different processes and also come about when both types of quenching occur. This combined quenching appears as an upward curve, and can be fitted to a second order polynomial:

F_0_/F= (1 + K_SV_ [Q])(1+ K_S_[Q]) = 1+ (K_SV_+ K_S_)[Q] + K_SV_K_S_[Q]^2^ (3)

**Figure 10:**
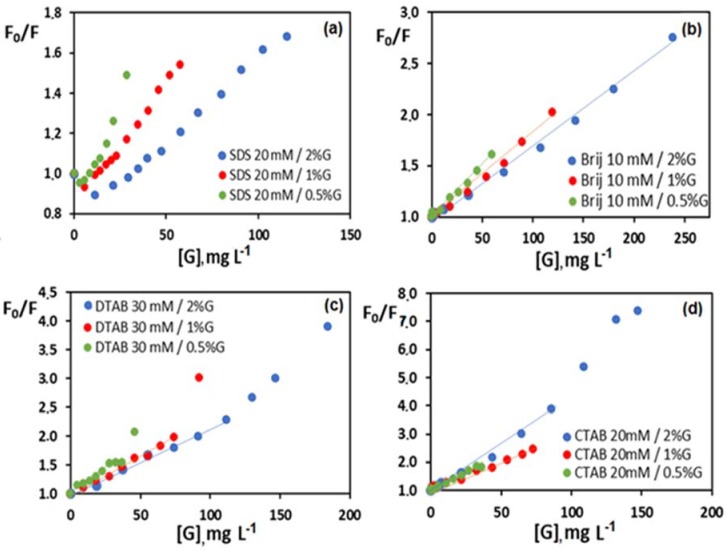
Ratio of the fluorescence intensity of vitamin B_6_ (λ_exc_ = 325 nm; λ_em_ = 395 nm) in the absence (F_o_) and in the presence (F) of G dispersed in: (**a**) 20 mM SDS; (**b**) 10 mM Brij L23; (**c**) 30 mM DTAB; and, (**d**) 20 mM CTAB vs. G concentration (mg L^−1^) for dispersions with a constant w_G_/w_S_.

To gain insight about the quenching process in the systems studied herein, F_0_/F ratio was plotted vs. the nanomaterial concentration for a constant w_G_/w_S_ (Figure 10), and the calculated quenching constants (either K_S_ or K_SV_, denoted by K) are displayed in Table 1. The quenching constants that were obtained for a constant surfactant concentration are compiled in Table S1 of the supplementary material.

For the ionic surfactants (Figure 10a,c,d), a linear tendency is observed at low G concentrations, which looses the linearity at high G loadings (i.e., 117, 111, and 87 mg L^−1^ for G (2 wt %) dispersions in SDS, DTAB, and CTAB, respectively). The upward curvature observed hints towards the coexistence of two or more quenching mechanisms. This behavior, which is characteristic of nanometer molecules, has been attributed to the formation of a complex in the ground state by means of electron transfer [6], and it has been described earlier for the attenuation of the fluorescence intensity of phthalocyanine dyes [34] and rhodamine 6G [35] by G. Nonetheless, the non-ionic surfactant (Figure 10b) shows a linear behavior in the whole concentration range studied, suggesting either a dynamic or a static quenching. The fact that the lineal interval of the plot F_0_/F vs. G is greater for the non-ionic surfactant when compared to the ionic ones corroborates the different mechanisms of interaction between the surfactant-wrapped G sheets and pyridoxine.

In the case of SDS dispersions (Figure 10a), it was not possible to fit the data to a straight line with intercept 1; hence, the K values for this system are not included in Table 1. Nonetheless, a linear relationship between F_0_/F and G concentration is found at low nanomaterial loadings, and the slope of the plot decreases with an increasing G/surfactant weight ratio, which hints towards a stronger pyridoxine-G interaction when the nanomaterial is more covered by the surfactant. This supports the hypothesis that, in this system, the key factors are the electrostatic attraction forces between G covered by the surfactant monomers and the vitamin in the zwitterionic form. These results differ from those that were reported for the quenching of riboflavin by G dispersions in SDS [23], where K values hardly changed with an increasing G percentage, corroborating the different mechanism of interaction between the vitamin and the G-wrapped flakes. Further, for riboflavin, a change in the slope of the plot F_0_/F vs. surfactant concentration was detected close to the CMC of SDS for the three G loadings that were investigated, implying a change in the quenching mechanism when micelles start to form.

Regarding G dispersions in Brij L23 (Figure 10b), a perfect relationship is found in the whole G concentration range (G ≤ 240 mg L^−1^), which is indicative of either static or a dynamic quenching, albeit lifetime measurements would be necessary to distinguish between [12]. A linear relation was also found for aromatic dyes [8,39], thrombin [40], and amino acids [41] in the presence of GO. On the other hand, K slightly decreases with an increasing G/surfactant weight ratio, albeit the difference between the three G percentages is small, which indicates hardly change in the strength of the interaction between the vitamin and the nanomaterial as the surfactant content is modified. This is consistent with the fact that in this system the essential is the π–π interactions between G (more or less covered by Brij) and pyridoxine, thus confirming the mere role of this surfactant as a dispersing agent. Interestingly, the values of K that were obtained are very close to those that were reported for the quenching of riboflavin by G dispersions in the same surfactant [23], where a linear relationship was also found in most of the concentration range, corroborating that for the non-ionic surfactant the key issues are the π–π stacking interactions between the electron-rich aromatic rings of vitamin B_2_ or B_6_ and the electron-acceptor rings of G.

The lineal interval for the dispersions in the cationic surfactants (Figure 10c,d) is smaller than that found for the non-ionic one, showing a clear positive deviation of the linearity at high G concentrations. Different theories have been proposed to explain this behaviour, including the formation of quencher-fluorophore complexes, or that the quenching reaction is very fast and diffusion controlled, while the Stern-Volmer equation assumes a steady state. Therefore, experimental data suggest the formation of pyridoxine-surfactant-wrapped G complexes, and they hint towards a combined mechanism of static and dynamic quenching, as what occurred with the fluorescence of vitamin B_2_ in the presence of polyethylene glycol (PEG) [11]. Another plausible explanation for the observed behavior could be the changes in the structure of the quenching agent [42]. Thus, the increase in the surfactant concentration could induce changes in the G surface that would modify the type of quenching. Nonetheless, it should be highlighted that the systems that were studied in this work are very complex, involving surfactant-vitamin, surfactant-G, and vitamin-G interactions, making it very difficult to elucidate the underlying quenching mechanisms.

In the case of DTAB, the change in the slope of the plot F vs. surfactant concentration (Figure 7a) takes place at about 18 mM, concentration slightly higher than its CMC, whilst for CTAB, the deviation occurs at about 10 times the CMC value (Figure 7b). These trends are in good agreement with those that were previously reported for the quenching of riboflavin in the presence of G dispersions in these cationic surfactants [23]. However, for pyridoxine, a slight change in K is found with increasing the G/surfactant weight ratio (Table 1), particularly for G dispersions in DTAB, suggesting a similar degree of interaction between DTAB and vitamin B_6_ for the three G percentages studied, whilst for riboflavin K, noticeably increased with an increasing G/DTAB weight ratio [23]. More interestingly, for vitamin B_2_, K values corresponding to G dispersions in DTAB were significantly higher than those that were calculated for pyridoxine, which is indicative of a stronger quenching, despite that the interaction between riboflavin and the DTAB-wrapped flakes is not electrostatic.

On the other hand, G dispersions in CTAB lead to the highest K values (Table 1), which is consistent with the strongest quenching effect on the fluorescence of pyridoxine when compared to the other surfactants. Further, the dispersion with 2% G shows a higher K value than those with a lower G percentage, which can be explained when considering its better degree of G exfoliation, as discussed earlier. An identical trend was found for K values that were obtained for dispersions prepared in a constant surfactant concentration (Table S1). These results are consistent with those found by Sarkar et al. [43] for the attenuation of the fluorescence intensity of 1-anthracene sulphonate by cationic alkyl trimethylammonium bromides; in such study, the K_SV_ values increased with decreasing the surfactant weight ratio, ascribed to the decrease in the quencher concentration in the bulk solution. It is worthy to note that the K value that was obtained for this dispersion (2% G in CTAB) is very similar to that reported for the fluorescence of vitamin E in the presence of the same amount of nanomaterial [12], and about twice that reported riboflavin [23].

## 4. Conclusions

A methodical comparative analysis of the effect of G dispersions in surfactants of different nature on the fluorescence of pyridoxine has been performed, and the results have been compared to those of a preceding study dealing with the quenching behavior of riboflavin. The surfactants act as G dispersing agents, and they efficiently exfoliate all of the G concentrations that were studied. In the absence of G, the fluorescence intensity of pyridoxine hardly changes with increasing the surfactant concentration. In the presence of G, a quenching phenomenon is found for all the systems, which in general becomes more pronounced with increasing the surfactant and G total concentrations, albeit it is independent on the G/surfactant weight ratio. For the same chain length, ionic surfactants are more effective than non-ionic ones in dispersing G, related with the different stabilization mechanisms in aqueous solutions. For the same G/surfactant weight ratio, the magnitude of the quenching effect follows the order: CTAB > DTAB > Brij ≥ SDS. For G dispersions in Brij L23, the experimental results fit to the Stern-Volmer equation in the whole G concentration range studied, suggesting either static or dynamic quenching. However, dispersions in the cationic surfactants show an upward deviation at higher loadings, which is indicative of a combination of both mechanisms. K values for G dispersions in the cationic surfactants are higher than those for the non-ionic one, particularly those in the surfactant with a higher molecular weight (CTAB), which is consistent with its most prominent quenching. In general, for a given G/ionic surfactant weight ratio, the surfactant-wrapped G flakes lead to a less pronounced quenching of riboflavin fluorescence when compared to pyridoxine, which is attributed to the weaker vitamin-surfactant interactions. The results that were obtained herein could be applied for the development of G-based fluorescence sensors for the determination of pyridoxine or riboflavin in complex systems.

## Figures and Tables

**Table 1:** Quenching constants (K) calculated from F_0_/F plots vs. G concentration for dispersions with a constant w_G_/w_S_ in the linear concentration range. In the case of SDS, it was not possible to attain a straight line with intercept 1.

Dispersion	[Surfactant] (mM)	[G] (mg L^−1^)	K (L mg^−1^)
30 mM DTAB/0.5% G	0–18	0–29	0.017 ± 0.008
30 mM DTAB/1% G	0–18	0–58	0.012 ± 0.004
30 mM DTAB/2% G	0–18	0–116	0.012 ± 0.002
20 mM CTAB/0.5% G	0–20	0–37	0.026 ± 0.002
20 mM CTAB/1% G	0–20	0–74	0.019 ± 0.002
20 mM CTAB/2% G	0–12	0–87	0.032 ± 0.006
10 mM Brij L23/0.5% G	0–10	0–60	0.010 ± 0.001
10 mM Brij L23/1% G	0–10	0–120	0.008 ± 0.001
10 mM Brij L23/2% G	0–10	0–240	0.0071 ± 0.0007

## Supplementary Materials

The following are available online at http://www.mdpi.com/1996-1944/11/6/888/s1, Figure S1: Raman spectrum of pristine graphene (G); Figure S2: SEM micrographs of pristine G at different magnifications: 2 µm (a); 1 µm (b,c); 0.5 µm (d); Figure S3: SEM images at different magnifications of G dispersions in 20 mM SDS: 0.5 wt % G (a–c); 1.0 wt % G (d–f); 2.0 wt % G (g–i); Figure S4: SEM images at different magnifications of G dispersions in 10 mM Brij L23: 0.5 wt % G (a–c); 1.0 wt % G (d–f); 2.0 wt % G (g–i); Figure S5: SEM images at different magnifications of G dispersions in 30 mM DTAB: 0.5 wt % G (a–c); 1.0 wt % G (d–f); 2.0 wt % G (g–i); Figure S6: SEM images at different magnifications of G dispersions in 20 mM CTAB: 0.5 wt % G (a–c); 1.0 wt % G (d–f); 2.0 wt % G (g–i); Table S1: Quenching constants (K) obtained from F_0_/F plots as a function of G concentration for G (0.5, 1 and 2 wt %) dispersions with a constant surfactant concentration.

## References

[1] Geim A.K., Novoselov K.S. (2007). The rise of graphene. Nat. Mater..

[2] Díez-Pascual A.M., Luceño Sánchez J.A., Peña Capilla R., García Díaz P. (2018). Recent advances in graphene/polymer nanocomposites for applications in polymer solar cells. Polymers.

[3] Diez-Pascual A.M., Diez-Vicente A.L. (2016). Poly(propylene fumarate)/polyethylene glycol-modified graphene oxide nanocomposites for tissue engineering. ACS Appl. Mater. Interfaces.

[4] Díez-Pascual A.M., Gómez-Fatou M.A., Ania F., Flores A. (2015). Nanoindentation in Polymer Nanocomposites. Prog. Mater. Sci..

[5] Salavagione H.J., Díez-Pascual A.M., Lázaro E., Vera S., Gomez-Fatou M.A. (2014). Chemical sensors based on polymer composites with carbon nanotubes and graphene: The role of the polymer. J. Mater. Chem. A.

[6] Lakowicz J.R. (2006). Principles of Fluorescence Spectroscopy.

[7] Pang Y., Cui Y., Ma Y., Qian H., Shen X. (2012). Fluorescence quenching of cationic organic dye by graphene: Interaction and its mechanism. Micro Nano Lett..

[8] Liu Y., Liu C.Y., Liu Y. (2011). Investigation on fluorescence quenching of dyes by graphite oxide and graphene. Appl. Surf. Sci..

[9] Lin W., Tian B., Zhuang P., Yin J., Zhang C., Li Q., Shih T., Cai W. (2016). Graphene based fluorescence-quenching-related fermi fevel elevation and electron concentration surge. Nano Lett..

[10] Vera-López S., Martínez P., San Andrés M.P., Díez-Pascual A.M., Valiente M. (2018). Study of graphene dispersions in sodium dodecylsulfate by steady-state fluorescence of pyrene. J. Coll. Interf. Sci..

[11] Díez-Pascual A.M., García-García D., San Andrés M.P., Vera S. (2016). Determination of riboflavin based on fluorescence quenching by graphene dispersions in polyethylene glycol. RSC Adv..

[12] San Andrés M.P., Díez-Pascual A.M., Palencia S., San Torcuato J., Valiente M., Vera S. (2017). Fluorescence quenching of α-tocopherol by graphene dispersed in aqueous surfactant solutions. J. Lumin..

[13] Díez-Pascual A.M., Ferreira C.H., Andrés M.P.S., Valiente M., Vera S. (2017). Effect of Graphene and Graphene Oxide Dispersions in Poloxamer-407 on the Fluorescence of Riboflavin: A Comparative Study. J. Phys. Chem. C.

[14] Salihoglu O., Kakenov N., Balci O., Balci S., Kocabas C. (2016). Graphene as a reversible and spectrally selective fluorescence quencher. Sci. Rep..

[15] Texter J. (2014). Graphene dispersions. Curr. Opin. Colloid Interface Sci..

[16] Wang S., Yi M., Shen Z. (2016). The effect of surfactants and their concentrations on the liquid-exfoliation of graphene. RSC Adv..

[17] McCormick D.B., Bowman B.A., Russell R.M. (1996). Vitamin B6. Present Knowledge in Nutrition (Volume 1).

[18] Dakshinamurti S., Dakshinamurti K., Zempleni J., Rucker R.B., Mc Cormick D.B., Suttie J.W. (2007). Vitamin B6. Handbook of Vitamins, 4th ed.

[19] Vanderschuren H., Boycheva S., Li K.T., Szydlowski N., Gruissem W., Fitzpatrick T.B. (2013). Strategies for vitamin B_6_ biofortification of plants: A dual role as a micronutrient and a stress protectant. Front. Plant Sci..

[20] Ahmad I., Mirza T., Qadeer K., Nazim U., Vaid F.H. (2013). Vitamin B_6_: Deficiency diseases and methods of analysis. Pak. J. Pharm. Sci..

[21] Garcia L., Blazquez S., San Andres M.P., Vera S. (2001). Determination of thiamine, riboflavin and pyridoxine in pharmaceuticals by synchronous fluorescence spectrometry in organized media. Anal. Chim. Acta.

[22] Metzler D.E., Snell E.E. (1955). Spectra and ionization constants of the vitamin B_6_ group and related 3-hydroxypyridine derivatives. J. Am. Chem. Soc..

[23] Mateos R., Vera S., Valiente M., Díez-Pascual A.M., San Andrés M.P. (2017). Comparison of anionic, cationic and nonionic surfactants as dispersing agents for graphene based on the fluorescence of riboflavin. Nanomaterials.

[24] Palencia S., Vera S., Díez-Pascual A.M., San Andrés M.P. (2015). Quenching of fluorene fluorescence by single-walled carbon nanotube dispersions with surfactants: Application for fluorene quantification in wastewater. Anal. Bioanal. Chem..

[25] Hao Y., Wang Y., Wang L., Ni Z., Wang Z., Wang R., Koo C.K., Shen Z., Thong J.T. (2010). Probing layer number and stacking order of few-layer graphene by Raman spectroscopy. Small.

[26] Ferrari A.C., Meyer J.C., Scardaci V., Casiraghi C., Lazzeri M., Mauri F., Piscanec S., Jiang D., Novoselov K.S., Roth S. (2006). Raman spectrum of graphene and graphene layers. Phys. Rev. Lett..

[27] Lotya M., King P.J., Khan U., De S., Coleman J.N. (2010). High-concentration, surfactant-stabilized graphene dispersions. ACS Nano.

[28] Shannigrahi M., Bagchi S. (2005). Novel fluorescent probe as aggregation predictor and micro-polarity reporter for micelles and mixed micelles. Spectrochim. Acta Part A.

[29] Hsieh A.G., Korkut S., Punckt C., Aksay I.A. (2013). Dispersion stability of functionalized graphene in aqueous sodium dodecyl sulfate solutions. Langmuir.

[30] Gou Q., Spada L., Vallejo-Lopez M., Lesarri A., Cocinero E.J., Caminati W. (2014). Interactions between alkanes and aromatic molecules: A rotational study of pyridine-methane. Phys. Chem. Chem. Phys..

[31] Kasry A., Ardakani A.A., Tulevski G.S., Menges B., Copel M., Vyklicky L. (2012). Highly efficient fluorescence quenching with graphene. J. Phys. Chem. C.

[32] Smith R.J., Lotya M., Coleman J.N. (2010). The importance of repulsive potential barriers for the dispersion of G using surfactants. New J. Phys..

[33] Meng W., Gall E., Ke F., Zeng Z., Kopchick B., Timsina R., Qiu X. (2015). Structure and interaction of graphene oxide-cetyltrimethylammonium bromide complexation. J. Phys. Chem. C.

[34] Zhang X.F., Shao X. (2014). π–π binding ability of different carbon nano-materials with aromatic phthalocyanine molecules: Comparison between graphene, graphene oxide and carbon nanotubes. J. Photochem. Photobiol. A.

[35] Bavali A., Parvin P., Mortazavi S.Z., Nourazar S.S. (2015). Laser induced fluorescence spectroscopy of various carbon nanostructures (GO, G and nanodiamond) in Rd6G solution. Biomed. Opt. Express.

[36] Wanless E.J., Ducker W.A. (1996). Organization of sodium dodecyl sulfate at the graphite-solution interface. J. Phys. Chem..

[37] Tummala N.R., Striolo A. (2008). Role of counterion condensation in the self-assembly of SDS surfactants at the water-graphite interface. J. Phys. Chem. B.

[38] Guardia L., Fernández-Merino M.J., Paredes J.I., Solís-Fernández P., Villar-Rodil S., Martínez-Alonso A., Tascón J.M.D. (2011). High-throughput production of pristine G in an aqueous dispersion assisted by non-ionic surfactants. Carbon.

[39] Wang X., He Y., Song G. (2012). A Graphene oxide-rhodamine 6G. Nanocomposite as turn on fluorescence probe for selective detection of DNA. Phys. Procedia.

[40] Dong H., Gao W., Yan F., Ji H., Ju H. (2010). Fluorescence resonance energy transfer between quantum dots and graphene oxide for sensing biomolecules. Anal. Chem..

[41] Li S. (August 2014). Analysis of the Interactions between Graphene Oxide and Biomolecules and Protein Fibrillation Using Surface Chemistry and Spectroscopy. Ph.D. Thesis.

[42] Ranjit S., Levitus M. (2012). Probing the interaction between fluorophores and DNA nucleotides by fluorescence correlation spectroscopy and fluorescence quenching. Photochem. Photobiol..

[43] Sarkar A., Pramanik S., Banerjee P., Bhattacharya S.C. (2008). Interaction of 1-anthracene sulphonate with cationic micelles of alkyl trimethyl ammonium bromides (CnTAB): A spectroscopic study. Colloids Surf. A.

