# Supplementary material for: Effect of Graphene Flakes Modified by Dispersion in Surfactant Solutions on the Fluorescence Behaviour of Pyridoxine
Source: Materials (Basel). 2018 May 25;11(6):888. doi: 10.3390/ma11060888 (PMC6025388; doi:10.3390/ma11060888)
Supplement: Supplementary file 1 [file materials-11-00888-s001.pdf]

Supplementary Materials

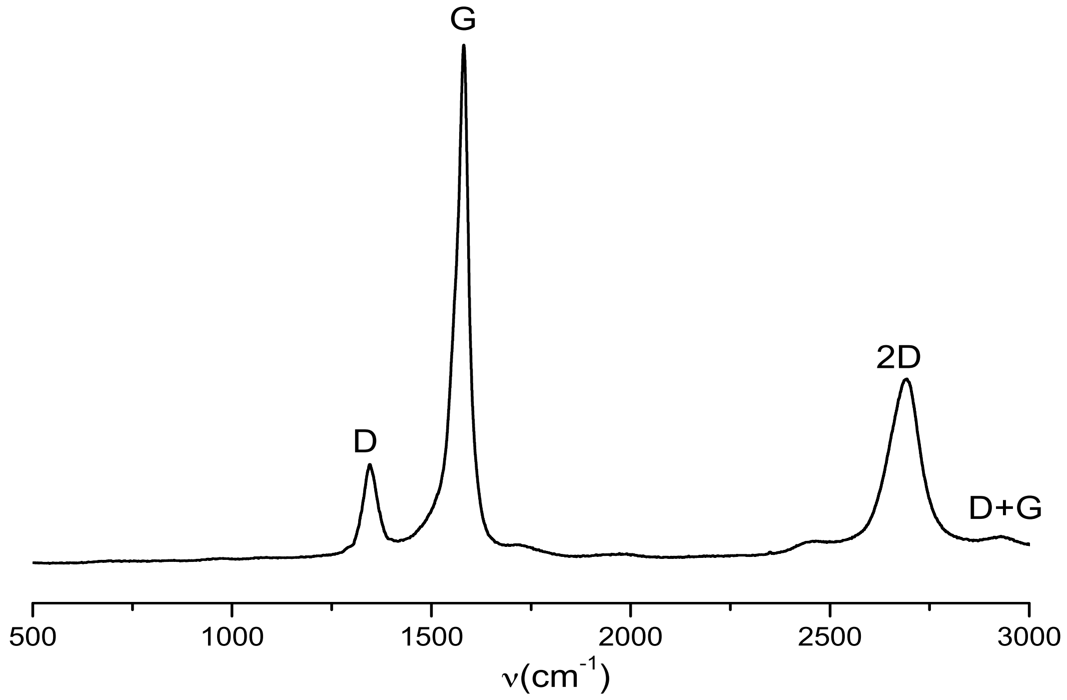

Figure S1. Raman spectrum of pristine graphene (G).

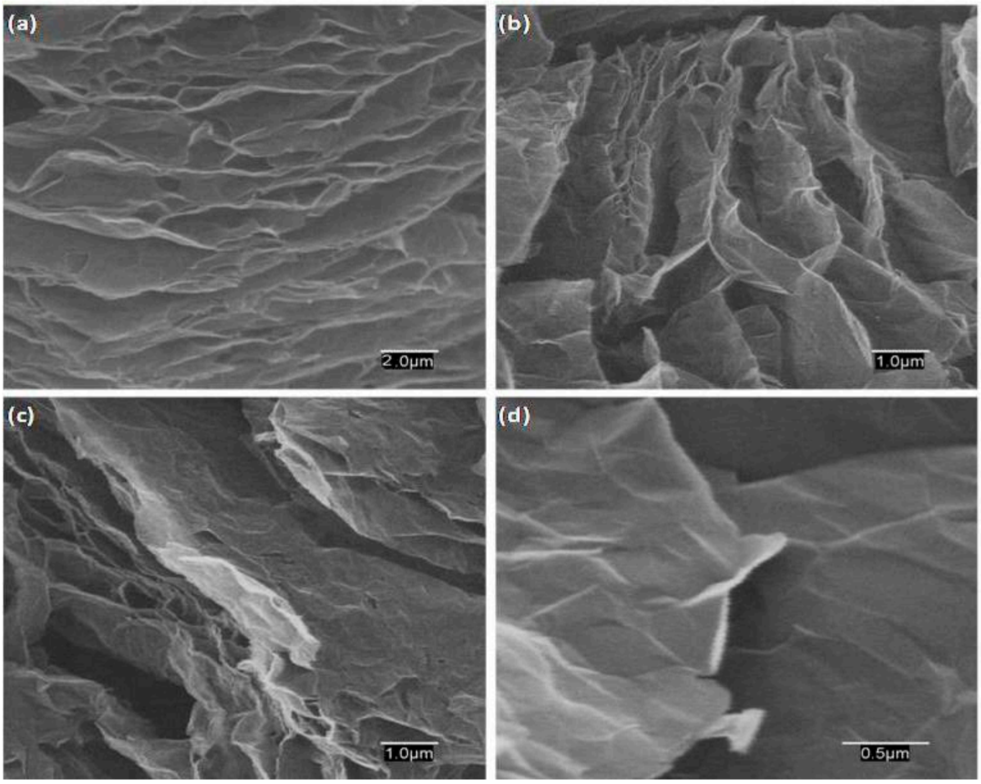

Figure S2. SEM micrographs of pristine G at different magnifications: 2 μm (**a**); 1 μm (**b** and **c**); 0.5 μm (**d**).

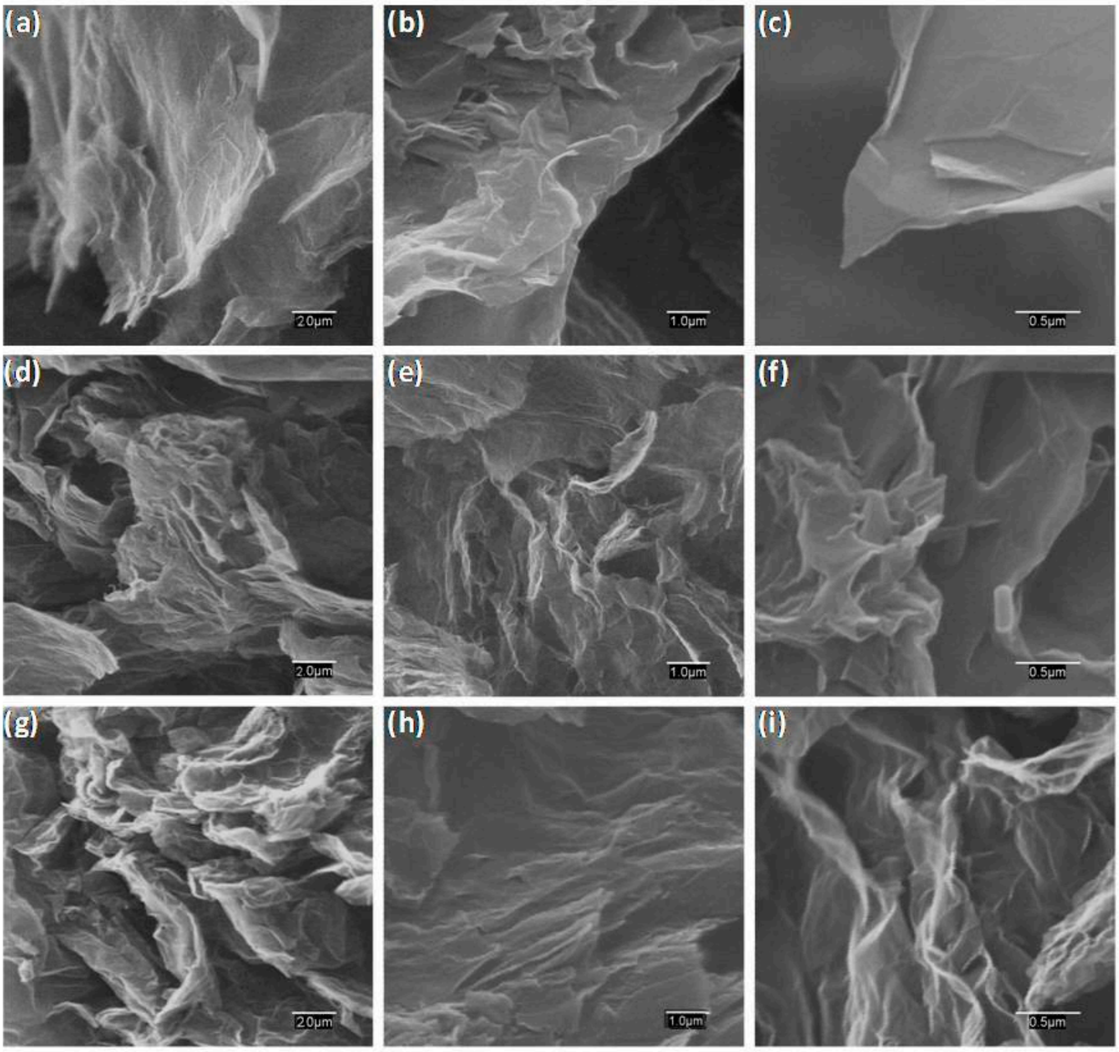

Figure S3. SEM images at different magnifications of G dispersions in 20 mM SDS: 0.5 wt % G (**a**, **b** and **c**); 1.0 wt % G (**d**, **e** and **f**); 2.0 wt % G (**g**, **h** and **i**).

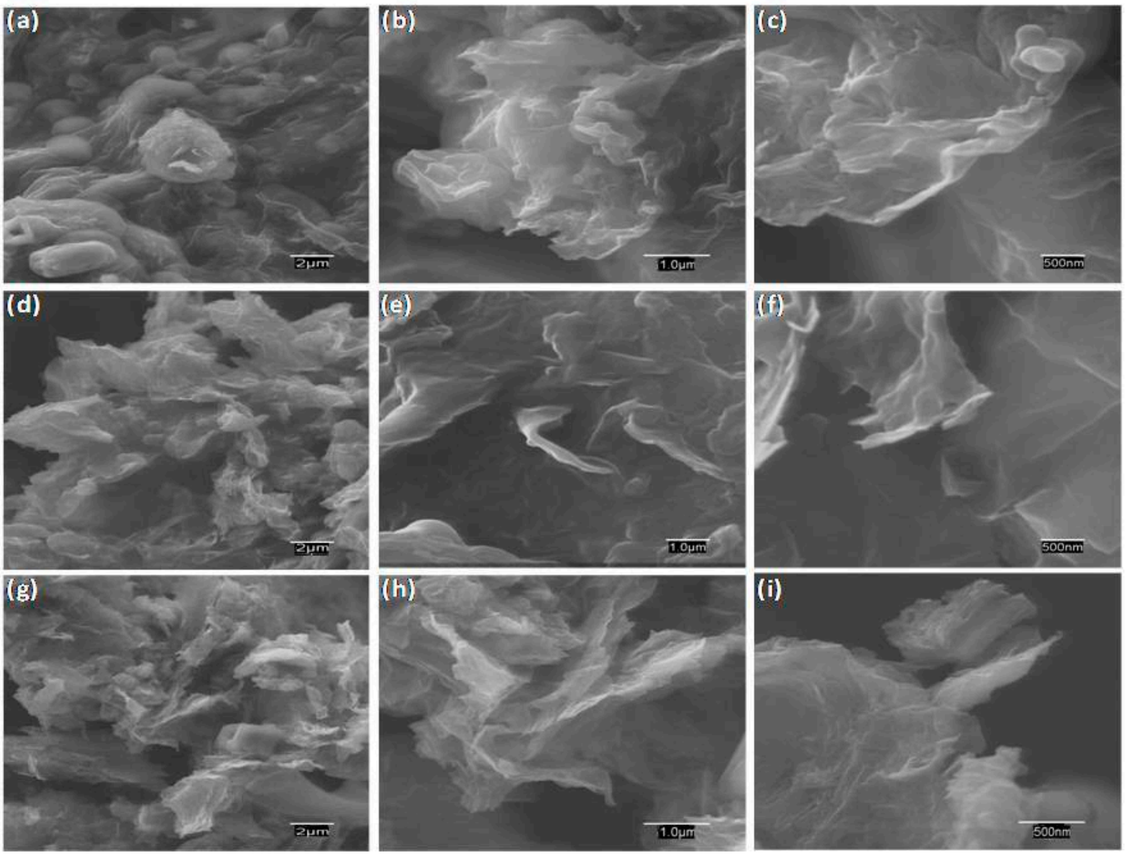

Figure S4. SEM images at different magnifications of G dispersions in 10 mM Brij L23: 0.5 wt % G (**a**, **b** and **c**); 1.0 wt % G (**d**, **e** and **f**); 2.0 wt % G (**g**, **h** and **i**).

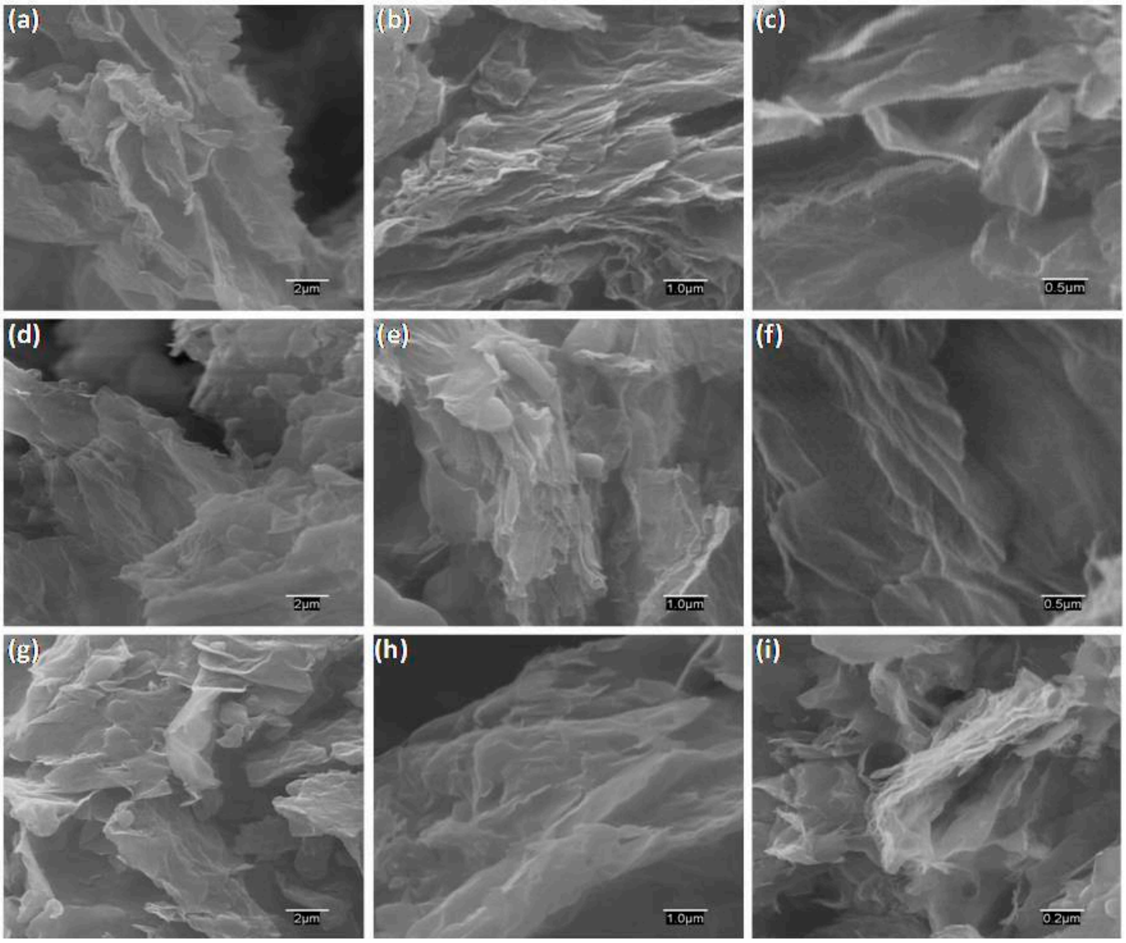

Figure S5. SEM images at different magnifications of G dispersions in 30 mM DTAB: 0.5 wt % G (**a**, **b** and **c**); 1.0 wt % G (**d**, **e** and **f**); 2.0 wt % G (**g**, **h** and **i**).

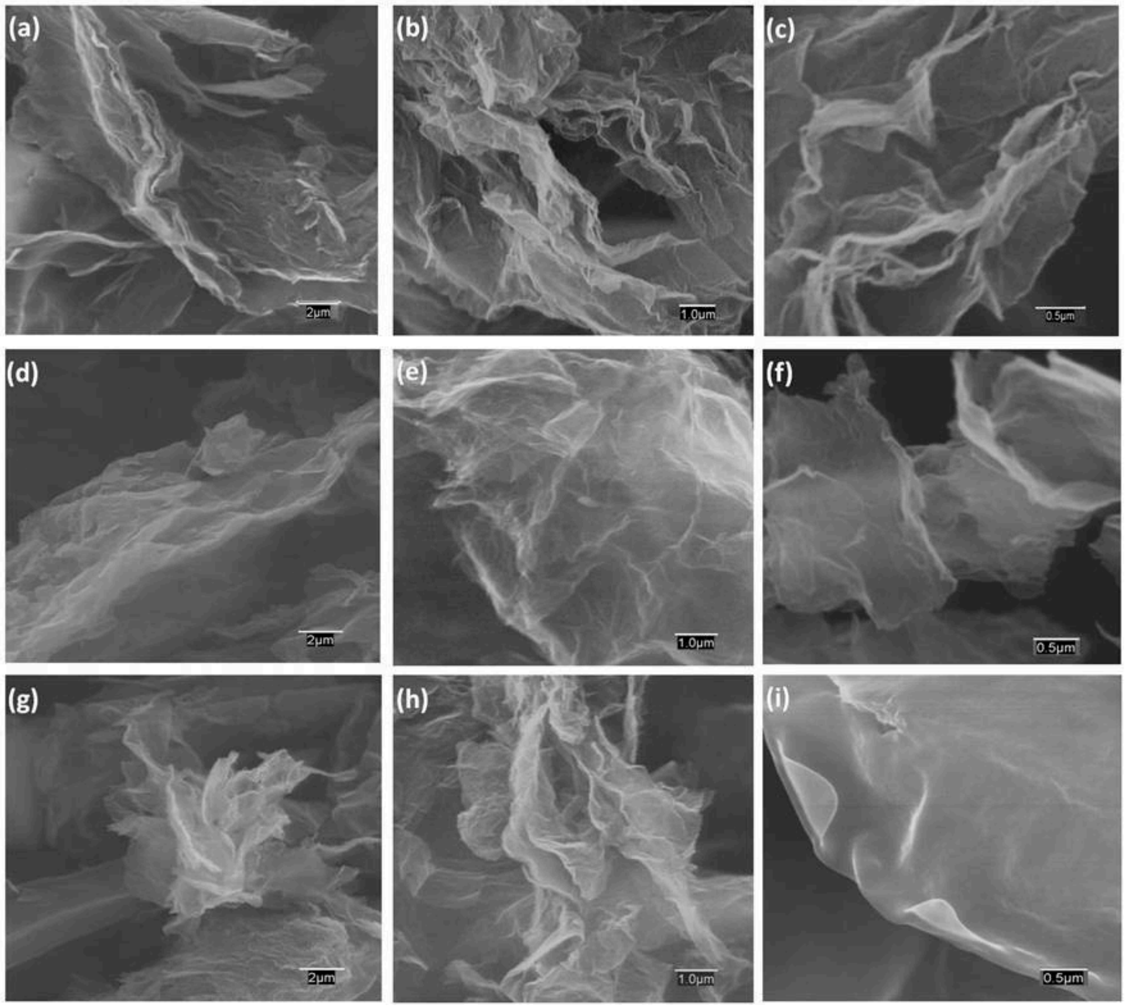

Figure S6. SEM images at different magnifications of G dispersions in 20 mM CTAB: 0.5 wt % G (**a**, **b** and **c**); 1.0 wt % G (**d**, **e** and **f**); 2.0 wt % G (**g**, **h** and **i**).

Table S1. Quenching constants (K) obtained from F_0/F plots as a function of G concentration for G (0.5, 1 and 2 wt %) dispersions with a constant surfactant concentration.

Dispersion	[G] (mg/L)	K (L/mg)
30 mM DTAB/ 0.5% G	0–29	0.019 ± 0.006
30 mM DTAB/ 1% G	0–58	0.014 ± 0.003
30 mM DTAB/ 2% G	0–116	0.012 ± 0.002
20 mM CTAB / 0.5% G	0–37	0.028 ± 0.002
20 mM CTAB / 1% G	0–74	0.020 ± 0.001
20 mM CTAB / 2% G	0–87	0.034 ± 0.003
10 mM Brij L23/ 0.5% G	0–60	0.0112 ± 0.0003
10 mM Brij L23/ 1% G	0–120	0.0059 ± 0.0004
10 mM Brij L23/ 2% G	0–240	0.0062 ± 0.0001
20 mM SDS / 0.5% G	0–29	0.013 ± 0.001
20 mM SDS / 1% G	0–58	0.015 ± 0.002
20 mM SDS / 2% G	0–116	0.019 ± 0.002
